# Thermoresponsive Polypeptide Fused L‐Asparaginase with Mitigated Immunogenicity and Enhanced Efficacy in Treating Hematologic Malignancies

**DOI:** 10.1002/advs.202300469

**Published:** 2023-06-04

**Authors:** Sanke Zhang, Yuanzi Sun, Longshuai Zhang, Fan Zhang, Weiping Gao

**Affiliations:** ^1^ Institute of Medical Technology Peking University Health Science Center Peking University School and Hospital of Stomatology Biomedical Engineering Department Peking University Peking University International Cancer Institute Peking University‐Yunnan Baiyao International Medical Research Center Beijing 100191 China

**Keywords:** artificial intelligence, elastin‐like polypeptide, hematological malignancies, immunogenicity, L‐asparaginase

## Abstract

L‐Asparaginase (ASP) is well‐known for its excellent efficacy in treating hematological malignancies. Unfortunately, the intrinsic shortcomings of ASP, namely high immunogenicity, severe toxicity, short half‐life, and poor stability, restrict its clinical usage. Poly(ethylene glycol) conjugation (PEGylation) of ASP is an effective strategy to address these issues, but it is not ideal in clinical applications due to complex chemical synthesis procedures, reduced ASP activity after conjugation, and pre‐existing anti‐PEG antibodies in humans. Herein, the authors genetically engineered an elastin‐like polypeptide (ELP)‐fused ASP (ASP‐ELP), a core‐shell structured tetramer predicted by AlphaFold2, to overcome the limitations of ASP and PEG‐ASP. Notably, the unique thermosensitivity of ASP‐ELP enables the in situ formation of a sustained‐release depot post‐injection with zero‐order release kinetics over a long time. The in vitro and in vivo studies reveal that ASP‐ELP possesses increased activity retention, improved stability, extended half‐life, mitigated immunogenicity, reduced toxicity, and enhanced efficacy compared to ASP and PEG‐ASP. Indeed, ASP‐ELP treatment in leukemia or lymphoma mouse models of cell line‐derived xenograft (CDX) shows potent anti‐cancer effects with significantly prolonged survival. The findings also indicate that artificial intelligence (AI)‐assisted genetic engineering is instructive in designing protein‐polypeptide conjugates and may pave the way to develop next‐generation biologics to enhance cancer treatment.

## Graphical abstract

Artificial intelligence is applied to design elastin‐like polypeptide (ELP) fused L‐asparaginase (ASP) (ASP‐ELP). ASP‐ELP features mitigated immunogenicity and toxicity, and enhanced pharmacokinetics and efficacy in treating hematologic malignancies. The unique thermosensitivity of ASP‐ELP leads to the in situ formation of a sustained release depot with zero‐order release kinetics, avoiding side effects associated with a transient release.

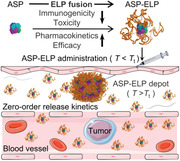

## 1 Introduction

Hematologic malignancies, mainly including leukemia, lymphoma, and multiple myeloma, are associated with poor prognosis and high mortality.^[^ ^1^ ^]^ In the past two decades, pharmaceutical development for hematologic malignancies has shifted from chemotherapy to biological therapeutics,^[^ ^2^ ^]^ such as monoclonal antibodies,^[^ ^3^ ^]^ immune checkpoint inhibitors,^[^ ^4^ ^]^ and chimeric antigen receptor T‐cells.^[^ ^5^ ^]^ However, these therapeutics have encountered formidable challenges due to off‐target effects,^[^ ^6^ ^]^ antigen escape,^[^ ^7^ ^]^ immune‐related adverse events,^[^ ^8^ ^]^ and immunotherapy resistance.^[^ ^9^ ^]^ L‐asparaginase (ASP), one of the most iconic therapeutics, has dominated the treatment of hematologic malignancies for more than 30 years.^[^ ^10^ ^]^ ASP can induce apoptosis of cancer cells by catalyzing the breakdown of L‐asparagine essential for their growth.^[^ ^11^ ^]^ Unfortunately, due to the high immunogenicity, severe toxicity, short half‐life, and poor stability of ASP, multiple high‐dose administrations are usually required to achieve adequate therapeutic efficacy, which inevitably induces severe side effects such as hypersensitivity reactions, pancreatitis, hepatic steatosis, cirrhosis, and coagulation disorders.^[^ ^12^ ^]^

PEGylation of ASP has been demonstrated to be an effective strategy to overcome these problems.^[^ ^13^ ^]^ Indeed, PEG‐ASP, commercially named Oncaspar, was approved by the Food and Drug Administration (FDA) as a first‐line treatment for acute lymphoblastic leukemia (ALL) in 2006.^[^ ^14^ ^]^ However, recent studies have found that pre‐existing anti‐PEG antibodies are widely present in humans due to the applications of PEG in food, cosmetics, and pharmaceuticals, which may induce accelerated blood clearance (ABC) phenomenon and severe hypersensitivity reactions, and reduce their clinical efficacy.^[^ ^15^ ^]^ Thus, researchers have attempted to conjugate poly(carboxy betaine) (PCB), glutamic acid and lysine (EK) peptides, or poly‐(L)‐proline (PLP) instead of PEG with ASP.^[^ ^16^ ^]^ Nevertheless, the inherent drawbacks of chemical synthesis, including complicated synthetic processes, lack of reproducibility, low yields, and high costs, render the clinical translation of those ASP‐polymer conjugates extremely difficult.^[^ ^17^ ^]^

**Figure 1:**
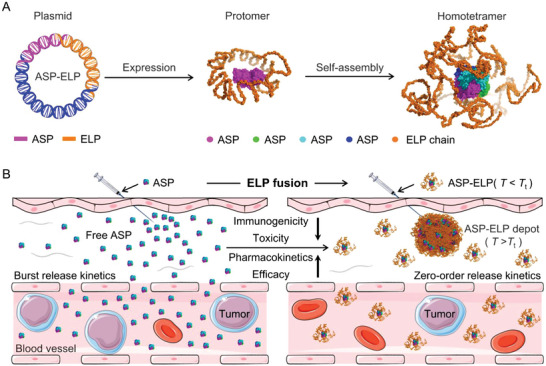
Schematic illustration of AI‐assisted design of a core‐shell structured tetramer of ASP‐ELP for treating hematological malignancies. A) Biosynthesis and structural prediction of ASP‐ELP. The ASP fragment was genetically fused to the N‐terminal of ELP to construct the ASP‐ELP plasmid, followed by overexpression in *Escherichia coli* (*E coli*). The homotetrameric complex structure was obtained using AlphaFold2 to predict the interaction between ASP‐ELP and ASP‐ELP. The ELP chains are wrapped around ASP's tetrameric surface to form a core‐shell structure that masks ASP's antigenic epitopes, which confers ASP‐ELP low immunogenicity and high stability. B) The unique thermosensitivity of ASP‐ELP makes it feasible to form a sustained release depot in situ with zero‐order release kinetics post‐injection, resulting in increased MTD, enhanced pharmacokinetics, mitigated immunogenicity, improved efficacy, and reduced toxicity. Conversely, the instantaneous distribution of highly immunogenic ASP into the systemic circulation system generates high‐concentration ASP that induces immunotoxicity and dose‐related toxicity, leading to poor pharmacokinetics and efficacy.

Elastin‐like polypeptides (ELPs) are a class of thermoresponsive, biodegradable, and biocompatible artificial polymers consisting of oligomeric repeats of a pentapeptide sequence, Val‐Pro‐Gly‐Xaa‐Gly, where the guest residue Xaa is any amino acid other than Pro.^[^ ^18^ ^]^ ELP changes from a monomeric state to aggregates above a transition temperature (*T*_t_), which is affected by the guest residue and chain length of ELP and its concentration. Our previous studies found that the ELP composed of Val‐Pro‐Gly‐Val‐Gly repeats can quickly form a sustained release depot with zero‐order release kinetics post‐injection, which prolonged drug half‐life, avoided high concentration‐induced side effects, raised maximum tolerated dose (MTD), and improved therapeutic efficacy of interferon‐alpha.^[^ ^19^ ^]^ Inspired by our previous findings, we hypothesized that ELP fusion of ASP would generate a core‐shell structured tetramer as predicted by AlphaFold2 to reduce ASP's immunogenicity by wrapping ASP's antigenic epitopes (**Figure** **1**A). We further reasoned that the unique thermosensitivity of ASP‐ELP should offer the formation of a sustained‐release drug depot upon intraperitoneal injection, leading to improved pharmacokinetics, reduced systemic toxicity, and enhanced antitumor efficacy (Figure 1B). To confirm whether ASP‐ELP can substitute PEG‐ASP and ASP as a more promising therapeutic agent for hematologic malignancies, we tested their performance by in vitro studies of activity retention and stability and in vivo studies of MTD, pharmacokinetics, immunogenicity, toxicity, and antitumor effects.

## 2 Results and Discussion

### 2.1 Structure Prediction of ASP‐ELPs by AlphaFold2

**Figure 2:**
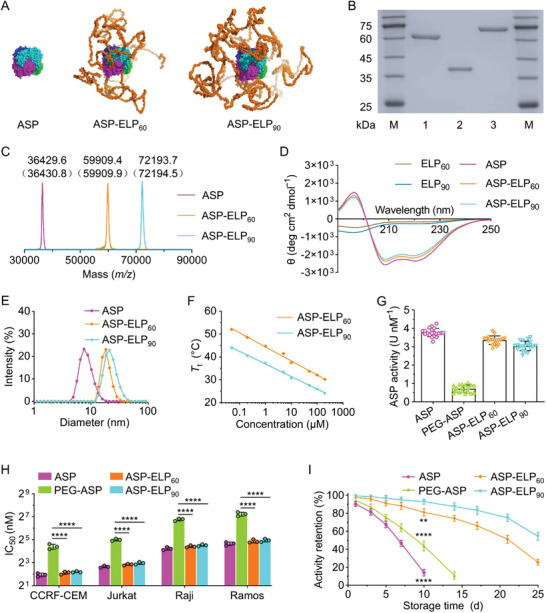
In vitro characterization of ASP‐ELPs. A) The predicted structures of the homotetrameric complexes were obtained by analyzing the interactions among ASP, ASP‐ELP_60_, and ASP‐ELP_90_ molecules by AlphaFold2. The ELP_90_ chains are more intensively wrapped around the ASP tetramer surface than the ELP_60_ chains. B) SDS‐PAGE confirmed the purity of ASP and ASP‐ELPs. Lane 1, ASP‐ELP_60_; lane 2, ASP; lane 3, ASP‐ELP_90_; lane M, Marker. C) The experimental molecular masses of ASP and ASP‐ELPs measured by MALDI‐TOF‐MS. The theoretical molecular masses calculated based on amino acid composition are in parentheses. D) The CD curves of ASP‐ELPs, ASP, and ELPs. E) DLS measurements indicated an increase in the hydrodynamic diameter of ASP‐ELPs compared to ASP. F) The *T*_t_ decreased with increasing concentration or ELP chain length. The transition temperature (*T*_t_) was defined as the turbidity versus temperature curve's inflection point and calculated as the first derivative's maximum. G) The enzyme activity of freshly prepared ASP, PEG‐ASP, and ASP‐ELPs. Data are mean ± SD (*n* = 20). H) In vitro cytotoxicity on human acute lymphoid leukemia cell lines (Jurkat and CCRF‐CEM) and human Burkitt's lymphoma cell lines (Raji and Ramos). Half‐maximum inhibitory concentration (IC_50_) was calculated with non‐linear fitting (sigmoidal curve fit) in Origin Pro 2021. For each cell line, the IC_50_ of PEG‐ASP was significantly higher than those of ASP‐ELPs. Data are mean ± SD (*n* = 3). I) Long‐term storage stability in 10 mM PBS at 37 °C. The activity retention (ratio of residual activity to initial activity) of ASP‐ELPs declined significantly slower with storage time than ASP and PEG‐ASP. Data are mean ± SD (*n* = 4). Statistical significance was determined by one‐way ANOVA followed by Tukey's multiple comparisons test in (H,I).

We conducted structural predictions of ASP‐ELPs with different chain lengths by the multimer model of Alphafold2 and selected two promising candidates, ASP‐ELP_60_ and ASP‐ELP_90_, for further study. ASP‐ELP_60_ and ASP‐ELP_90_ form a core‐shell homotetrameric complex, where their ASP molecules form a tetrameric core, similar to ASP's tetrameric structure, while flexible, randomly coiled ELP chains are wrapped around their ASP's cores, forming a shell‐like structure (**Figure** **2**A). The generally low predicted local distance difference test (plDDT) score of the ELP chains confirmed the structural disorder.^[^ ^20^ ^]^ We found that the longer ELP chains can sufficiently wrap the ASP tetramer surface to reduce the exposure of epitopes, leading to a greater reduction in immunogenicity. Considering the two‐sided effects of ELP on ASP, that is, both blocking ASP's immunogenic sites and reducing its bioactivity, we performed a series of in vitro and in vivo experiments to more accurately assess the effect of ASP‐ELP_60_ and ASP‐ELP_90_ on enhancing ASP's performance.

### 2.2 Biosynthesis and Physicochemical Properties of ASP‐ELPs

We utilized coomassie‐stained sodium dodecyl sulfate‐polyacrylamide gel electrophoresis (SDS‐PAGE) to monitor the overexpression in *Escherichia coli* and the purification by inverse transition cycling (ITC) of ASP‐ELPs (Figure S1, Supporting Information) and to confirm the purity of ASP and ASP‐ELPs (Figure 2B). Matrix‐assisted laser desorption/ionization‐time of flight mass spectrometry (MALDI‐TOF‐MS) further substantiated that the molecular masses of purified ASP and ASP‐ELPs were approximately equivalent to the theoretical values (Figure 2C). The secondary structure of ASP and ASP‐ELPs detected by circular dichroism (CD) was utterly identical, indicating that the ELP chains did not affect ASP's structure (Figure 2D), as predicted by AlphaFold2. The hydrodynamic diameters of ASP‐ELP_60_ and ASP‐ELP_90_ measured by dynamic light scattering (DLS) were 18.6 and 22.5 nm, which were 2.2 and 2.7 times that of ASP (8.4 nm), respectively (Figure 2E), implying that ASP‐ELPs would have longer half‐lives than ASP as their larger sizes lead to reduced renal clearance. As expected, ASP‐ELPs were thermoresponsive with a sharp transition from the soluble to the coacervate state when the physical temperature exceeded their phase transition temperature (*T*_t_) (Figure S2, Supporting Information). Herein the *T*_t_ is an inflection point in the turbidity versus temperature curve, which decreased with increasing ASP‐ELP concentration and ELP chain length (Figure 2F).

### 2.3 Enhanced In Vitro Activity Retention of ASP‐ELPs

We assayed the activity of freshly prepared ASP, PEG‐ASP, ASP‐ELP_60_, and ASP‐ELP_90_ with Nessler's reagent to be 3.8, 0.7, 3.4, and 3.1 U nM^−1^, respectively (Figure 2G). ASP‐ELP_60_ and ASP‐ELP_90_ acquired high levels of activity retentions of 89% and 81% compared to unmodified ASP, which were 4.7 and 4.3 times that of PEG‐ASP. The high activity retentions of ASP‐ELPs over PEG‐ASP could arise from the procedural simplicity and structural precision of biosynthesis. The slightly lower activity of ASP‐ELP_90_ over ASP‐ELP_60_ likely arises from the higher coverage level of the ELP chain over ASP in ASP‐ELP_90_, as predicted by AlphaFold2 (Figure 2A).

We further performed cytotoxicity assays on Jurkat, CCRF‐CEM, Raji, and Ramos cells and obtained half‐maximal inhibitory concentrations (IC_50_) for each ASP analog on each cell line (Figure S3, Supporting Information). As anticipated, the IC_50_s of ASP‐ELPs were significantly lower than that of PEG‐ASP for each cell line, indicating that ASP‐ELPs have more potent cytotoxicity due to the higher activity retention (Figure 2H).

### 2.4 Improved In Vitro Stability of ASP‐ELPs

To compare long‐term storage stability, we measured the enzyme activity of ASP, PEG‐ASP, ASP‐ELP_60_, or ASP‐ELP_90_ stored in 10 mM PBS at 37 °C for different periods using Nessler's reagent (Figure 2I). ASP‐ELP_90_ showed the best storage stability with the slowest activity loss with time, and both ASP‐ELP conjugates outperformed ASP and PEG‐ASP. For example, after 10 days of storage, the remaining activity was 93% for ASP‐ELP_90_ and 81% for ASP‐ELP_60_, which was significantly higher than that of ASP at 14% and PEG‐ASP at 44%. Notably, after 25 days of storage, ASP‐ELP_90_ and ASP‐ELP_60_ still retained 54.5% and 25.6% of initial activity, respectively. We attribute the higher storage stability of ASP‐ELPs to the protective effects of the ELP chains on ASP. ASP‐ELP_90_ is stabler than ASP‐ELP_60_, likely caused by the longer ELP chain providing more adequate packaging and protection.

### 2.5 Increased MTDs of ASP‐ELPs

We determined the MTDs of ASP, PEG‐ASP, ASP‐ELP_60_, and ASP‐ELP_90_ by dose‐escalation trials on healthy BALB/c mice (Figure S4, Supporting Information). ASP‐ELP_90_’s MTD was 2400 U kg^−1^ body weight (BW), which was 4.8, 3.4, and 1.3 times that of ASP's 500 U kg^−1^ BW, ASP‐PEG's 700 U kg^−1^ BW, and ASP‐ELP_60_’s 1800 U kg^−1^ BW, respectively. We attributed the significant increase in MTDs of ASP‐ELPs over ASP and PEG‐ASP to the following reasons: i) the concentration‐dependent thermosensitivity of ASP‐ELPs would contribute to the formation of sustained‐release depots, avoiding high concentration‐induced side effects associated with burst release; ii) the core‐shell structured tetramer of ASP‐ELPs would confer low immunogenicity to ASP‐ELPs, leading to insignificant immune‐related side effects.

### 2.6 Sustained Release Depots of ASP‐ELPs

**Figure 3:**
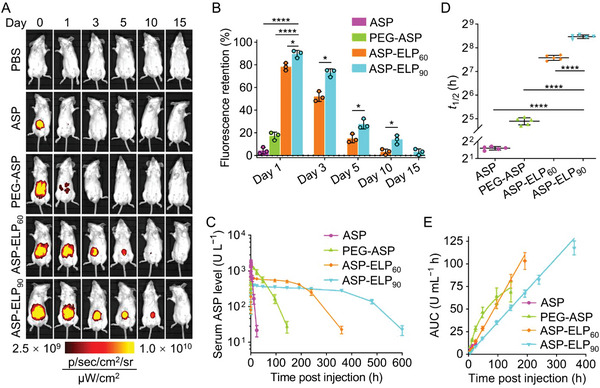
ASP‐ELPs featured sustained release and improved pharmacokinetics. A) Representative images of the fluorescence intensity variation of Cy5‐labeled ASP, PEG‐ASP, ASP‐ELP_60_, or ASP‐ELP_90_ at the injection site (*n*  =  3). B) ASP‐ELP_90_ possessed a higher fluorescence retention (ratio of residual fluorescence to initial fluorescence) at the injection site than ASP, PEG‐ASP, and ASP‐ELP_60_. Data are mean ± SD (*n*  =  3). Statistical difference was determined using an unpaired two‐tailed Student's *t*‐test. C) Serum ASP activity levels of mice in each group at defined time points upon injection with the MTDs of ASP, PEG‐ASP, ASP‐ELP_60_, and ASP‐ELP_90_. Data are mean ± SD (*n* = 5). D) The *t*_1/2_ values are calculated by fitting the databases to a one‐compartment mode. Data are mean ± SD (*n* = 5). Statistical significance was determined by one‐way ANOVA followed by Tukey's multiple comparisons test. E) The AUCs of ASP‐ELP_90_ and ASP‐ELP_60_ were linearly correlated with time, while the AUCs of PEG‐ASP and ASP were logarithmically correlated with time. Data are mean ± SD (*n* = 5).

To elucidate the vivo release process, we monitored the fluorescence intensity variation of Cy5‐labeled ASP, PEG‐ASP, ASP‐ELP_60_, or ASP‐ELP_90_ at their MTDs using in vivo imaging system (IVIS). Just as hypothesized, Cy5‐ASP‐ELPs formed fluorescent depots post‐injection and their fluorescent signals gradually declined with time, while the fluorescent signals of Cy5‐ASP and Cy5‐PEG‐ASP rapidly vanished from the injection sites (**Figure** **3**A). Specifically, after 1 day, the fluorescence retention at the injection site (ratio of residual fluorescence to initial fluorescence) remained high for Cy5‐ASP‐ELP_90_ (91%) and Cy5‐ASP‐ELP_60_ (78%), while that for PEG‐ASP significantly reduced to 17% and fluorescence for ASP almost vanished (4%) (Figure 3B). Interestingly, on day 10, the fluorescence retention of Cy5‐ASP‐ELP_90_ was 4.1‐fold higher than that of Cy5‐ASP‐ELP_60_. ASP‐ELPs can form sustained‐release depots post‐injection due to the body temperature higher than *T*_t_ and a large local concentration (Figure 2F). Moreover, ASP‐ELP_90_, which has a lower *T*_t_ compared to ASP‐ELP_60_ due to its longer ELP chain length, forms a more stable depot with slower fluorescence declination. The decrease of fluorescence with time at the injection site is due to the gradual dispersion of soluble ASP‐ELP out of the depot, which provides a subtle strategy to avoid the burst‐release of drugs and increase the circulation half‐time.

### 2.7 Improved Pharmacokinetics of ASP‐ELPs

We tested serum ASP activity levels of ASP analogs post‐injection at their MTDs (Figure 3C) and acquired primary pharmacokinetic parameters. The significantly lower peak concentrations (*C_max_*) of ASP‐ELP_90_ (404 U L^−1^) and ASP‐ELP_60_ (661 U L^−1^) than PEG‐ASP (1444 U L^−1^) and ASP (1766 U L^−1^) implied a reduction in the high‐concentration induced side effects (Figure S5, Supporting Information). The terminal half‐life (*t*_1/2_) of ASP‐ELP_90_ (353.2 h) was 1.8‐, 11.9‐, and 116.8‐fold longer than that of ASP‐ELP_60_ (191.0 h), ASP‐PEG (29.8 h), or ASP (3.0 h) (Figure 3D), indicating a more durable therapeutic period of ASP‐ELP conjugates, especially ASP‐ELP_90_. Notably, the area under the curves (AUC) for ASP‐ELP_90_ over 360 h and ASP‐ELP_60_ over 192 h were linearly correlated with time (Figure 3E), indicating zero‐order release kinetics. In contrast, the AUCs of ASP‐PEG and ASP were logarithmically associated with time, revealing abrupt release kinetics which could induce severe high‐concentration‐associated side effects. Additionally, ASP‐ELP_90_ is more prominent than ASP‐ELP_60_ in improving pharmacokinetics due to the longer zero‐order release time caused by the lower *T*_t_.

### 2.8 Mitigated Immunogenicity of ASP‐ELPs

**Figure 4:**
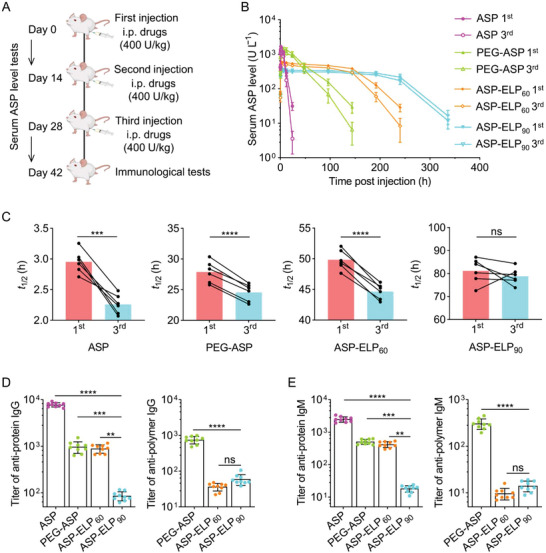
ASP‐ELP_90_ possessed lower immunogenicity compared with ASP, PEG‐ASP, and ASP‐ELP_60_. A) Schematic illustration of the primary events of the immunogenicity assay. The “i.p.” represents “intraperitoneal injection.” Blood samples were collected at given time points after the first and third injections to measure ASP activity levels. Mice were euthanized on day 42, and blood samples were collected and tested for immunogenicity. B) Serum ASP activity levels in ASP, PEG‐ASP, and ASP‐ELP_60_ groups were lower after the third injection than after the first injection. Data are represented as mean ± SD (*n*  =  6). C) The *t*_1/2_ values in ASP, PEG‐ASP, and ASP‐ELP_60_ groups were significantly lower after the third injection than after the first injection, while in the ASP‐ELP_90_ group, no significant changes were observed after the two injections. Data are represented as mean ± SD (*n*  =  6). Statistical difference was determined using a paired two‐tailed Student's *t*‐test. D,E) The titers of anti‐protein IgG/IgM and anti‐polymer IgG/IgM in ASP, PEG‐ASP, ASP‐ELP_60_, and ASP‐ELP_90_ groups. The anti‐protein IgG/IgM titers of ASP‐ELP_90_ were lower than those of ASP‐ELP_60_, PEG‐ASP, and ASP. There were no significant differences in anti‐ELP IgG and IgM titers between ASP‐ELP_90_ and ASP‐ELP_60_, whereas the anti‐PEG IgG/IgM titers of PEG‐ASP were higher than the anti‐ELP IgG/IgM titers of ASP‐ELPs. Data are mean ± SD (*n* = 9). Statistical significance was determined by one‐way ANOVA followed by Tukey's multiple comparisons test.

Based on the predicted structures of ASP, ASP‐ELP_60_, and ASP‐ELP_90_ using AlphaFold2, we speculated that ASP‐ELP conjugates reduce ASP's immunogenicity by wrapping the immunogenic sites on the surface of ASP, and ASP‐ELP_90_ would be less immunogenic than ASP‐ELP_60_ as the ELP_90_ chains wrapped ASP's surface more adequately (Figure 2A). To verify our assumption, we performed an immunogenicity assay by conducting multiple injections of a specific ASP conjugate in mice (**Figure** **4**A). We found that serum ASP activity levels and *t*_1/2_ in the ASP, PEG‐ASP, and ASP‐ELP_60_ groups were lower after the third injection than after the first injection. In contrast, serum ASP activity levels and *t*_1/2_ were almost equivalent in the ASP‐ELP_90_ group after both injections (Figure 4B,C). In short, these pharmacokinetic data exhibited that the accelerated blood clearance (ABC) phenomenon of ASP‐ELP_90_ was insignificant compared to ASP, PEG‐ASP, and ASP‐ELP_60_.

As expected, the anti‐protein immunoglobulin G (IgG)/immunoglobulin M (IgM) titers in serum after injecting ASP‐ELP_60_, PEG‐ASP, and ASP were 10‐/23‐, 11‐/28‐ and 90‐/137‐fold higher than those of ASP‐ELP_90_, respectively (Figure 4D,E), confirming the remarkably reduced anti‐ASP immunogenicity of ASP‐ELP_90_. We also measured the anti‐polymer IgG/IgM titers to further clarify our findings (Figure 4D,E). The IgG/IgM titers of PEG‐ASP against the PEG polymer were 21‐/31‐ and 13‐/22‐ times higher than those of ASP‐ELP_60_ against ELP_60_ and ASP‐ELP_90_ against ELP_90_, respectively, indicating that the immunogenicity of ELP was much lower than that of PEG. Additionally, IgG/IgM titers against ELP were not significantly different between the ASP‐ELP_90_ and ASP‐ELP_60_ groups, demonstrating the immunogenicity of ELP is independent of the ELP chain length.

Collectively, these results indicate that the ELP_90_ chains are more effective in obscuring ASP's antigenic epitopes than PEG and the ELP_60_ chains, just as predicted by AlphaFold2.

### 2.9 Reduced Systemic Toxicity of ASP‐ELPs

**Figure 5:**
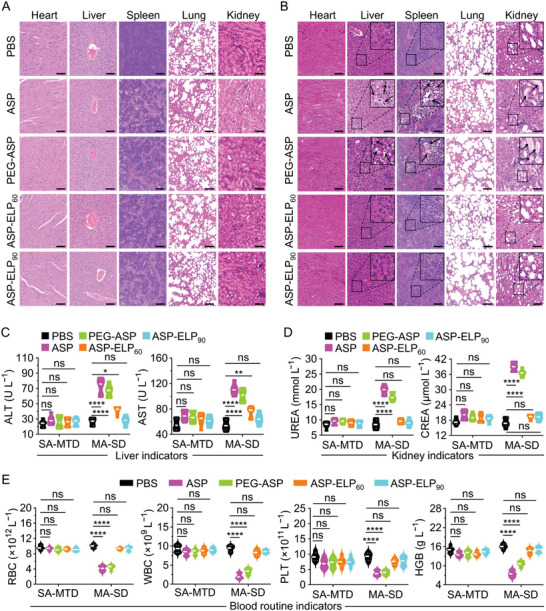
Toxicity assessments after two dosing regimens (SA‐MTD and MA‐SD). The “SA‐MTD” represents “a single administration of the maximum tolerated dose”; and the “MA‐SD” represents “multiple administrations of the same dose”. A) Representative images of H&E‐stained pathological sections after SA‐MTD. No apparent histopathological alterations were observed (*n*  =  6). Scale bar, 100 µm. B) Representative images of H&E‐stained pathological sections after MA‐SD (*n*  =  6). The lipid vacuoles due to hepatocyte degeneration in liver tissues (black arrows) indicated liver injury; swollen, degenerated, and necrotic splenocytes in spleen tissues (black arrows) demonstrated severe splenic injury; and homogeneous red‐stained proteinaceous depots and detached necrotic renal tubular epithelial cells in kidney tissues (black arrows) suggested acute tubular interstitial injury. Scale bar, 100 µm. C) Liver indicator analysis. Significantly elevated AST and ALT indicated ASP‐induced liver injury or abnormal liver function (*n*  =  6). D) Kidney indicator analysis. Markedly raised CREA and BUN suggested ASP‐induced renal failure or abnormal renal function (*n*  =  6). E) Blood routine indicator analysis. Decreased RBCs, WBCs, PLTs, and HGB suggested the possibility of hematopoietic suppression (*n*  =  6). Data in (C–E) are mean ± SD (*n* = 6), and the statistical difference was determined by one‐way ANOVA followed by Tukey's multiple comparisons test.

We evaluated two dosing regimens in our in vivo toxicity studies: a single administration of the maximum tolerated dose (SA‐MTD) and multiple administrations of the same dose (MA‐SD). More specifically, SA‐MTD was the single administration of 500 U kg^−1^ BW for ASP, 700 U kg^−1^ BW for PEG‐ASP, 1800 U kg^−1^ BW for ASP‐ELP_60_, and 2400 U kg^−1^ BW for ASP‐ELP_90_; MA‐SD was six administrations of 400 U kg^−1^ BW per week. For the toxicity assessment in SA‐MTD, no apparent histopathological alterations and no abnormal hematological indicators were found in all treatment groups compared with the PBS group (**Figure** **5**A). However, for the toxicity assessment in MA‐SD, we observed apparent histologic lesions in Hematoxylin and Eosin (H&E) pathological sections of the liver, spleen, and kidney from the ASP and PEG‐ASP groups (Figure 5B). Consistent with histopathological findings, several hematological parameters were abnormal in the ASP and PEG‐ASP groups, including liver indicators of aspartate aminotransferase (AST) and alanine aminotransferase (ALT) (Figure 5C), kidney indicators of creatinine (CREA) and blood urea nitrogen (BUN) (Figure 5D), and blood routine indicators of red blood cells (RBCs), white blood cells (WBCs), platelets (PLTs), and hemoglobin (HGB) (Figure 5E). We attributed the toxic side effects in ASP and PEG‐ASP groups to immune‐related toxicity induced by high‐immunogenic ASP and PEG‐ASP following six injections and to dose‐related toxicity induced by high peak activity concentrations following each injection. As expected, there were no significant histological abnormalities and no statistically significant differences in hematological indicators in the ASP‐ELP_60_ and ASP‐ELP_90_ groups relative to the PBS group, except that ALT and AST were significantly elevated in the ASP‐ELP_60_ group. The superior in vivo safety of ASP‐ELP_90_ compared to ASP‐ELP_60_ is due to its lower immunogenicity and peak concentration. In conclusion, the excellent performances of ASP‐ELP_90_ in reducing the incidence of toxic side effects support it as a potential clinical candidate to replace ASP and PEG‐ASP.

### 2.10 Enhanced Antitumor Efficacy of ASP‐ELPs

**Figure 6:**
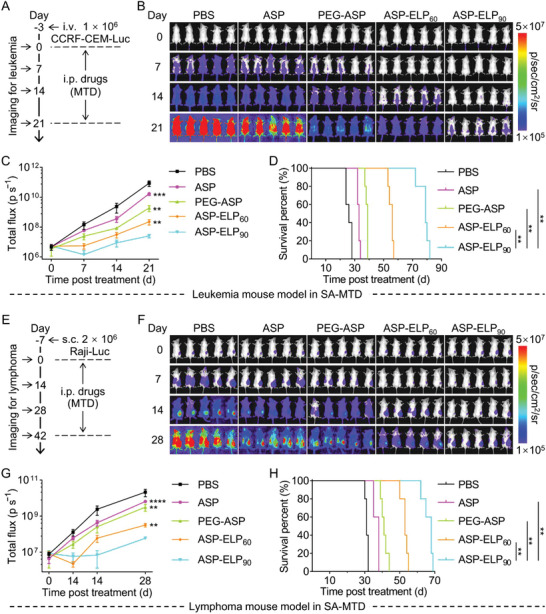
ASP‐ELP_90_ outperformed ASP, PEG‐ASP, and ASP‐ELP_60_ in anticancer efficacy after SA‐MTD in CDX mouse models of A—D) CCRF‐CEM‐Luc leukemia and E—H) Raji–Luc lymphoma. The “SA‐MTD” represents “a single administration of the maximum tolerated dose”. The “i.p.” represents “intraperitoneal injection”; the “i.v.” represents “intravenous injection”; and the “s.c.” represents “subcutaneous injection”. A,E) Schematic illustration of the primary events of the anti‐leukemia or anti‐lymphoma assay. B,F) Bioluminescence images of mice harboring CCRF‐CEM‐Luc or Raji–Luc cells in SA‐MTD (*n* = 5). C,G) Bioluminescence signal over time in mice carrying CCRF‐CEM‐Luc or Raji–Luc. Data are mean ± SD (*n* = 5 per group). Statistical difference was determined using an unpaired two‐tailed Student's *t*‐test. D,H) Survival curves of mice carrying CCRF‐CEM‐Luc or Raji–Luc cells upon pharmacological treatment with PBS, ASP, PEG‐ASP, ASP‐ELP_60_, and ASP‐ELP_90_ (*n* = 5 per group). Survival significance was assessed by a log‐rank (Mantel–Cox) test.

**Figure 7:**
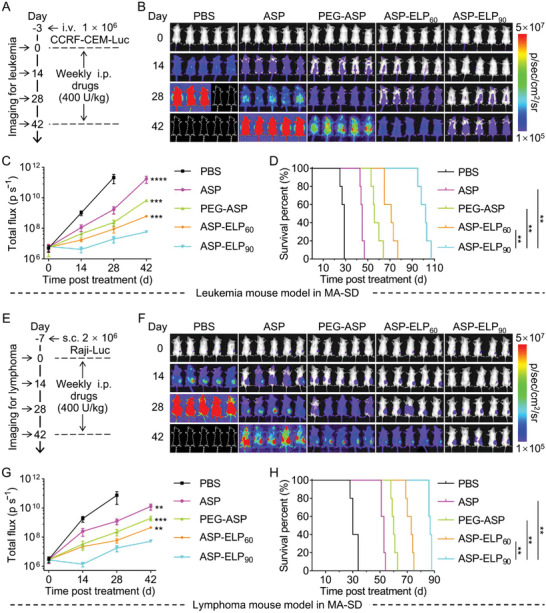
ASP‐ELP_90_ outperformed ASP, PEG‐ASP, and ASP‐ELP_60_ in anticancer efficacy after MA‐SD in CDX mouse models of A—D) CCRF‐CEM‐Luc leukemia and E—H) Raji–Luc lymphoma. The “MA‐SD” represents “multiple administrations of the same dose”. A,E) Schematic illustration of the primary events of the anti‐leukemia or anti‐lymphoma assay. B,F) Bioluminescence images of mice harboring CCRF‐CEM‐Luc or Raji–Luc cells in MA‐SD (*n* = 5). The white dashed line represents the natural death of post‐treatment mice. C,G) Bioluminescence signal over time in mice carrying CCRF‐CEM‐Luc or Raji–Luc. Data are mean ± SD (*n* = 5 per group). Statistical difference was determined using an unpaired two‐tailed Student's *t*‐test. D,H) Survival curves of mice carrying CCRF‐CEM‐Luc or Raji–Luc cells upon treatment with PBS, ASP, PEG‐ASP, ASP‐ELP_60_, and ASP‐ELP_90_ (*n* = 5 per group). Survival significance was assessed by a log‐rank (Mantel–Cox) test.

To evaluate the antitumor efficacy of ASP, PEG‐ASP, ASP‐ELP_60_, and ASP‐ELP_90_, two treatment regimens of SA‐MTD (**Figure** **6**) and MA‐SD (**Figure** **7**) were evaluated in leukemia or lymphoma mouse models of cell line‐derived xenograft (CDX), respectively. For the leukemia mouse model in SA‐MTD, the in vivo leukemia proliferation was obviously slower in the ASP‐ELP_90_ group than ASP, PEG‐ASP, and ASP‐ELP_60_ groups (Figure 6B,C). Meanwhile, the median survival time of mice in the ASP‐ELP_90_ group was 1.25‐, 1.79‐, 2.12‐, and 2.6‐folds longer than that in the ASP‐ELP_60_, PEG‐ASP, ASP, and PBS groups, respectively (Figure 6D). Similar results were observed for the lymphoma mouse model in SA‐MTD (Figure 6F–H). These results indicate that despite a single administration, ASP‐ELP_90_ prolongs cancer remission more significantly than ASP, PEG‐ASP, and ASP‐ELP_60_.

We further evaluated the anti‐leukemic and anti‐lymphoma efficacy in MA‐SD, ASP‐ELP_90_ group also exhibited more potent antitumor efficacy and significantly longer survival times than ASP‐ELP_60_, ASP‐PEG, and ASP groups (Figure 7). The results above show that ASP‐ELP_90_ is markedly more effective in treating hematological cancers than ASP, PEG‐ASP, and ASP‐ELP_60_ at the same dosing regimen. In conclusion, ASP‐ELP_90_, a more effective therapeutic agent, is promising as a substitute for ASP and PEG‐ASP for treating hematological malignancies.

## 3 Discussion

In this study, we innovatively fused ELP to ASP with the assistance of AlphaFold2 and revealed multiple benefits of the designed ASP‐ELPs over ASP and PEGylated ASP (trade name Oncaspar) in treating leukemia and lymphoma. This work shows that fusing ASP with ELP significantly outperforms PEG conjugation in in vitro enzyme activity and stability, and in vivo immunogenicity, biosafety, and treating efficacy. For pharmacokinetics and systemic side effects, our study demonstrates that thermosensitive ASP‐ELPs can form sustained‐release depots post‐injection to show platform‐to‐trough fluctuations in blood, avoiding the high concentration‐induced side effects of ASP and PEGylated ASP. Notably, we found that the immunogenicity of ASP‐ELP_90_ is over one order of magnitude lower than PEG‐ASP, indicating that the immunogenicity of ASP is more effectively reduced by using ELP chains than PEG chains to wrap the antigenic epitopes of ASP. Owing to no apparent dose‐related and immune‐related side effects, mice treated with ASP‐ELPs can tolerate higher doses without significant toxicity relative to ASP and PEGylated ASP. As a result of the above significant advantages, ASP‐ELPs exhibit enhanced efficacy in treating leukemia and lymphoma as compared to ASP and PEGylated ASP.

We can rationally design ASP‐ELPs with desirable structures and functions based on high‐accuracy structure prediction of AlphaFold2, improving drug development efficiency. To our knowledge, AI has not been applied to design protein‐polymer conjugates to accelerate drug development. Two drug candidates, ASP‐ELP_60_ and ASP‐ELP_90_, were selected based on the promising levels of chain coverage calculated by AlphaFold2 and were experimentally tested for their properties and efficacies. Indeed, these two candidates both outperform PEG‐ASP and ASP as confirmed by experimental approaches. Interestingly, even though ASP‐ELP_90_, the one with higher chain coverage over ASP, processes slightly lower enzyme activity, its storage stability, MTD, pharmacokinetics, immunogenicity, biosafety, and treatment efficacy are all superior to ASP‐ELP_60_ and is identified as the most promising drug candidate in this work. Overall, our studies demonstrate a novel and efficient therapeutic agent ASP‐ELP_90_ for treating hematological malignancies and AI‐assisted genetic engineering of protein‐polypeptide conjugates as a promising strategy for creating next‐generation therapeutics.

## 4 Experimental Section

### Mice

Eight‐week‐old female BALB/c and NOD/SCID mice with a weight of 19–20 g were obtained from Beijing Vital River Laboratory Animal Technology Co. Ltd (Beijing, China). Mice were bred and maintained in a specific pathogen‐free environment at the Peking University Institute of Systems Biomedicine. All animal experiments were performed in accordance with relevant guidelines and regulations, and animal experimental protocols (Approval No. LA2022205) were approved by the Institutional Animal Care and Use Committee at Peking University.

### Biosynthesis of ASP, ASP‐ELP60, and ASP‐ELP90

The gene encoding ASP (UniProtKB/Swiss‐Prot: P00805.2) was amplified by Polymerase Chain Reaction (PCR) and inserted into the pET‐25b (+) vector. Recursive directional ligation by plasmid reconstruction (RE‐RDL) was employed for recombinant plasmids encoding ELP_60_ and ELP_90_ genes.^[^ ^21^ ^]^ PCR‐amplified ASP fragments, together with ELP_60_ or ELP_90_ were inserted into the pET‐25b (+) vector to construct recombinant plasmids (ASP‐ELP_60_ or ASP‐ELP_90_), respectively. Following overexpression in *E. coli* Rosetta (DE3) cells, ASP‐ELPs and ELPs were purified by inverse transition cycling (ITC),^[^ ^22^ ^]^ and ASP was purified by nickel column affinity chromatography (GE Healthcare, America). Expression and purification of ASP, ASP‐ELP_60_, and ASP‐ELP_90_ are available in the supplementary material of this article. The purified proteins were subsequently passed through the Endotoxin Removing Gel (Thermo Scientific, America) to remove bacterial endotoxins, freeze‐dried, and stored at ‐80 °C. Purity was assessed at each purification step through sodium dodecyl sulfate‐polyacrylamide gel electrophoresis (SDS‐PAGE).

### Thermoresponsive Characteristics

The phase transition behavior of ASP‐ELPs was assessed by analyzing the optical density (OD) at 350 nm (OD_350_) versus the temperature of sample solutions on a SpectraMax M3 microplate reader (Molecular Devices, America). A series of diluted samples were added to a transparent 96‐well plate (200 µL per well), and their OD_350_ values were recorded at corresponding temperatures from 10 to 60 °C.

### Cytotoxicity Assay

10 µL of ASP, PEG‐ASP, ASP‐ELP_60_, and ASP‐ELP_90_ were added to 96‐well plates containing 100 µL cell suspension (5000 cells per well) at final concentrations of 0.5, 1, 2.5, 5, 10, 25, 50, 75, 100, 250, 500, or 1000 nM. After 48 h, 10 µL of CCK‐8 solution was added to each well and incubated for 1.5 h. Each well's OD_450_ was measured using a SpectraMax M3 microplate reader. Experiments were repeated three times with three independent wells (*n* = 9). Below is a formula for calculating cell viability (%): (OD_sample_ − OD_background_)/(OD_control_ − OD_background_) ×100%. The half‐maximal inhibitory concentration (IC_50_) was obtained by fitting dose‐effect curves to monotonic sigmoidal models, resulting in the dose at which cell survival was 50%.

### Sustained Release Assay

BALB/c mice were injected intraperitoneally with Cy5‐labeled ASP, PEG‐ASP, ASP‐ELP_60_, or ASP‐ELP_90_ at their MTDs, or an equal volume of PBS as a control (*n* = 3 for each group). After mice were anesthetized with 1.25% of 2,2,2‐tribromoethanol at the dose of 20 µL g^−1^ BW, the sedated mice were imaged at the specified time points using an IVIS Imaging System 200 Series (Caliper Life Sciences). The fluorescence intensity of the injection site was measured using Living Image 4.5 software.

### Pharmacokinetics

ASP, PEG‐ASP, ASP‐ELP_60_, and ASP‐ELP_90_ were injected intraperitoneally in BALB/c mice at their MTDs (*n* = 5 for each group). Blood samples were collected from the tail vein at the specified time to measure serum ASP activity levels using an Asparaginase Activity Assay Kit (Biovision, America). Pharmacokinetic parameters were calculated using a one‐compartment model by the Pharmacokinetic simulation software Drug and Statistics 2.0.

### Immunogenicity Analysis

Biweekly for 6 weeks, BALB/c mice were injected intraperitoneally with ASP, PEG‐ASP, ASP‐ELP_60_, or ASP‐ELP_90_ at 400 U kg^−1^ BW or an equivalent volume of PBS (*n* = 9 for each group). After the first and third injections, blood samples from the same six mice were collected at the indicated time points to measure serum ASP activity levels. On day 42, the mice were euthanized with the blood samples collected and tested for immunogenicity (*n* = 9). 100 µL ASP, PEG‐ASP, ASP‐ELP_60_, or ASP‐ELP_90_ (0.1 mg mL^−1^) were placed into a high binding capacity clear 96‐well plates (Tymora, Japan) and incubated at 4 °C overnight for antigen encapsulation. All wells were cleaned with PBS and blocked with the blocking buffer (pH 8.0, 0.1 M tris buffer, 1% nonfat milk) for 2 h. All wells were then washed five times repeatedly using PBS, filled with 100 µL of serum samples from BALB/c mice diluted by PBS, and incubated at 37 °C for 2 h. The plates were washed five times repeatedly with PBS. Next, 100 µL of horseradish peroxidase (HRP)‐conjugated goat anti‐mouse immunoglobulin G (IgG)/immunoglobulin M (IgM) secondary antibody was added and incubated for 1 h at 37 °C to enable the antibody to bind to the antigen. After being washed five times using PBS, all wells were incubated with 100 µL of 3,3′,5,5′‐tetramethylbenzidine for the chromogenic reaction. The reactions were terminated after 15 min by adding 100 µL H_2_SO_4_ (0.2 M). Each well's OD_450_ was recorded with a SpectraMax M3 microplate reader. For the measurement of anti‐ELP_60_, anti‐ELP_90_, and anti‐PEG antibody levels, ELP_60_, ELP_90,_ and PEG‐IFN conjugates were used as antigens in the encapsulation process.

### Safety Assessment

On day 42, blood samples (*n* = 6 for each group) and tissue samples (heart, liver, spleen, lung, and kidney) from a random mouse among six mice were obtained for hematological and histopathological analysis in two dosing regimens (SA‐MTD and MA‐SD). Red blood cells (RBCs), white blood cells (WBCs), platelets (PLTs), and hemoglobin (HGB) were acquired using a Celltac *α* MEK‐6450 automatic hematology analysis (NihonKohden, Japan). Aspartate aminotransferase (AST), alanine aminotransferase (ALT), creatinine (CREA), and blood urea nitrogen (UREA) were analyzed with a Hitachi 7600 automatic analyzer (Hitachi, Japan). The fresh tissue blocks (not exceeding 0.5 cm in thickness) were fixed overnight in 4% neutral paraformaldehyde and embedded in paraffin wax for histopathological evaluation. Paraffin sections were imaged using a VECTRA Automated Quantitative Pathology Imaging System (PerkinElmer, America) after being stained with hematoxylin and eosin.

### Antitumor Efficacy

1 × 10^6^ CCRF‐CEM‐Luc cells were inoculated into NOD/SCID via the tail vein to construct a CDX leukemia mouse model and 1 × 10^6^ Raji–Luc cells were injected subcutaneously into the dorsal side of NOD/SCID mice to construct a CDX lymphoma mouse model. After pharmacological treatment with PBS, ASP, PEG‐ASP, ASP‐ELP_60_, or ASP‐ELP_90_ (*n* = 5 for each group of each regimen), anesthetized mice carrying CCRF‐CEM‐Luc or Raji–Luc cells were injected with dibenzotriazine (0.3 µM per mouse) for imaging and monitored for in vivo tumor burden with the IVIS imaging system.

### Statistical Analysis

Statistics and graphs were generated using GraphPad Prism 9 or Origin Pro 2021. Statistics are calculated using a two‐tailed paired or unpaired Student's *t*‐test, one‐way ANOVA followed by Tukey's multiple comparisons test or log‐rank (Mantel–Cox) test. Indicators of statistical significance are no significant (ns) *p* > 0.05, *p < 0.05, **p < 0.01, ***p < 0.001, and ****p < 0.0001.

## Conflict of Interest

The authors declare no conflict of interest.

## Author Contributions

W.G. conceived and supervised the project. S.Z. and W.G. analyzed the data; S.Z. performed the experiments; L.Z. and F.Z. participated in animal experiments; W.G. wrote the manuscript; W.G., Y.S., and S.Z. revised the paper; All authors proofread the manuscript.

## Supporting information

Supporting Information

## Data Availability

The data that support the findings of this study are available from the corresponding author upon reasonable request.
