# Supplementary material for: Thermoresponsive Polypeptide Fused L‐Asparaginase with Mitigated Immunogenicity and Enhanced Efficacy in Treating Hematologic Malignancies
Source: Adv Sci (Weinh). 2023 Jun 4;10(23):2300469. doi: 10.1002/advs.202300469 (PMC10427413; doi:10.1002/advs.202300469)
Supplement: Supplementary file 1 — Supporting Information [file ADVS-10-2300469-s001.pdf]

ADVANCED SCIENCE
Open Access

Supporting Information

for *Adv. Sci.*, DOI 10.1002/advs.202300469

Thermoresponsive Polypeptide Fused L-Asparaginase with Mitigated Immunogenicity and Enhanced Efficacy in Treating Hematologic Malignancies

*Sanke Zhang, Yuanzi Sun, Longshuai Zhang, Fan Zhang and Weiping Gao**

Supporting Information

Thermoresponsive Polypeptide Fused L-Asparaginase with Mitigated Immunogenicity and Enhanced Efficacy in Treating Hematologic Malignancies

Sanke Zhang, Yuanzi Sun, Longshuai Zhang, Fan Zhang, and Weiping Gao*

S. Zhang, Y. Sun, L. Zhang, F. Zhang, and Prof. W. Gao
Institute of Medical Technology, Peking University Health Science Center, Peking University School and Hospital of Stomatology, Biomedical Engineering Department, Peking University, Peking University International Cancer Institute, Peking University-Yunnan Baiyao International Medical Research Center, Beijing 100191, China.
*E-mail: gaoweiping@hsc.pku.edu.cn

1. Experimental Section

Materials: Molecular biology reagents, chemical reagents, and cell culture reagents were purchased from New England Biolabs, Sigma-Aldrich, and Gibco, respectively, unless otherwise specified.

Cells: Human acute lymphatic leukemia cell lines (Jurkat and CCRF-CEM) and human Burkitt's lymphoma cell lines (Raji and Ramos) were purchased from the Cell Bank of the Chinese Academy of Science (Shanghai, China). Luciferase-expressing CCRF-CEM (CCRF-CEM-Luc) and luciferase-expressing Raji (Raji-Luc) cell lines were obtained from Meisen Chinese Tissue Culture Collections (Zhejiang, China). We cultured Jurkat, CCRF-CEM, Raji, Ramos, CCRF-CEM-Luc, and Raji-Luc cells in RPMI 1640 containing 10% FBS and 1% streptomycin/penicillin at 37 °C in a 5% CO_2 incubator.

Expression and Purification of ASP, ASP-ELP$_{60}$, and ASP-ELP$_{90}$: After gene verification by DNA Sequencing, the plasmids carrying ASP, ASP-ELP$_{60}$, or ASP-ELP$_{90}$ genes were respectively transformed into *E. coli* Rosetta-gami (DE3) pLys Chemical Competent Cells. Cells harboring the desirable plasmid were selected on Agar plates by the specific antibiotic resistance, which was ampicillin for ASP and kanamycin for ASP-ELP conjugates. The corresponding antibiotics were present at 50 μg mL^{-1} in all media used in the following steps. The selected cell colonies were grown in 5-10 mL Luria-Bertani medium before being

transferred into 1 L terrific broth (TB) medium and cultured at 37 °C, 220 rpm until the optical density at 600 nm (OD_{600}) reached 0.8. After the temperature was adjusted to 20 °C, protein expression was induced with 0.5 mM isopropyl-ß-d-thiogalactopyranoside (IPTG). The induced cells were incubated overnight and then harvested by centrifugation at 12000 rpm for 15 min. The cell pellets were suspended in 10 mM pre-cooled PBS and lysed using a science-IID ultrasonic processor (Ningbo Xinzhi, China). 10% (w/v) polyethyleneimine (PEI) was used to precipitate nucleic acids in the lysate and the supernatant after centrifugation was used for further purification.

The ELP fusion proteins were purified by three rounds of inverse transition cycling (ITC).[1] In each round, phase transition was triggered by incubating the sample at 37 °C for 15 min in the presence of 2 M NaCl. The aggregated ASP-ELPs were separated from the solutions by centrifugation at 14000 rpm for 10 min at 37 °C and redissolved in pre-cooled PBS. Insoluble impurities were pelleted after centrifugation at 4 °C and discarded.

ASP was purified by nickel affinity chromatography. The supernatant containing ASP was filtered through a 0.45-µm-pore-size filter (Millipore, USA) and applied to a pre-equilibrated HisTrap HP 5 mL column (GE Healthcare, USA) mounted on an AKTA Purifier system (GE Healthcare, USA). ASP with a His_6 tag was eluted using 20% Buffer A (0 mM HEPES, 500 mM NaCl, 5 mM imidazole, pH 7.4) and 80% Buffer B (20 mM HEPES, 500 mM NaCl, 500 mM imidazole, pH 7.4). A desalting step was then performed using a HiPrep 26/10 Desalting Column (GE Healthcare, USA).

Physicochemical Characteristics: Molecular masses of ASP, ASP-ELP_{60}, and ASP-ELP_{90} were measured by MALDI-TOF-MS (AB SCIEX TOF/TOF 5800, Applied Biosystems) using sterile water-dissolved protein solutions. Hydrodynamic diameters were measured by a Zetasizer Nano ZS90 DLS system (Malvern, UK) at 10 °C using protein solutions dissolved in 10 mM PBS. Secondary structures were monitored with a J-1500 CD spectrometer (Jasco International, Tokyo, Japan) in the wavelength range of 250 nm to 190 nm. The obtained spectral data are converted to mean molar residual ellipticity and smoothed by Origin Pro 2021.

Activity Assay: The enzymatic activity of freshly prepared ASP, PEG-ASP, ASP-ELP_{60}, and ASP-ELP_{90} was detected using Nessler's reagent (Merck, Germany). A mixture of 50 mM L-Asparagine (L-Asn) and 10 nM enzyme in 50 mM Tris–HCl were incubated at 37 °C for 10 min, followed by sequential additions of reaction-terminating trichloroacetic acid and

Nessler's reagent. The absorbance of ammonium sulfate at 410 nm monitored on a SpectraMax Paradigm microplate reader (Molecular Devices, America) was used to estimate enzyme activity. One unit of ASP activity is defined as the amount of enzyme that produces 1.0 μM urea per minute at 37 °C.

To quantify storage stability, the enzymatic activities of ASP, PEG-ASP, ASP-ELP$_{60}$, and ASP-ELP$_{90}$ were measured using Nessler's reagent after being stored at 37 °C in 10 mM PBS for 1, 3, 5, 7, 10, 14, 17, 21, and 25 days.

Maximum Tolerated Dose (MTD): BALB/c mice were intraperitoneally injected with ASP at the doses of 300, 500, 700, or 900 U kg^{-1} body weight (BW), PEG-ASP at 500, 700, 900, or 1100 U kg^{-1} BW, ASP-ELP$_{60}$ at 1600, 1800, 2000, or 2200 U kg^{-1} BW, or ASP-ELP$_{90}$ at 2200, 2400, 2600, or 2800 U kg^{-1} BW (n = 3 for each dose of each drug). Mice survival and body weight change were monitored daily for two weeks. The MTD was defined as the maximum activity dose tested which didn't result in the death of any animal or a body weight loss of more than 10%.

2. Supplemental Figures

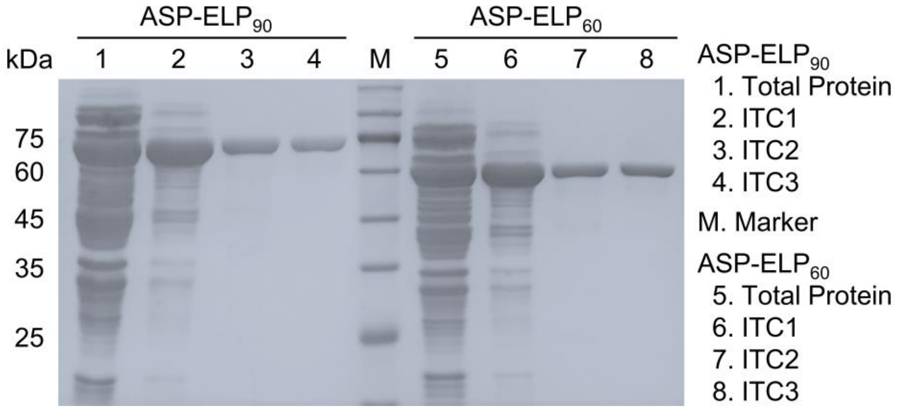

Figure S1. The purification of ASP-ELP$_{60}$ and ASP-ELP$_{90}$ by ITC was monitored by SDS-PAGE.

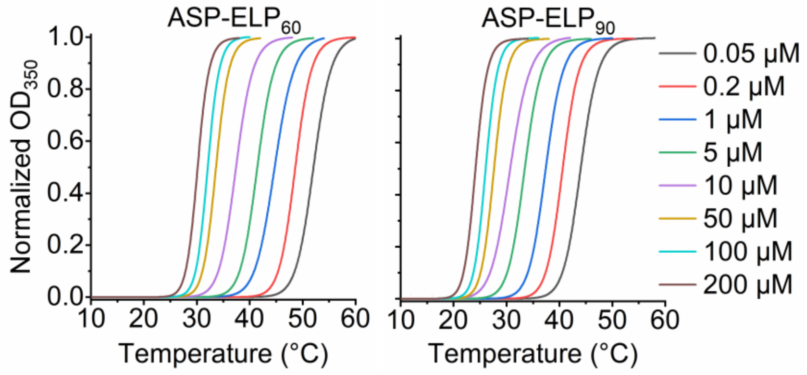

Figure S2. The phase transition behaviors at different concentrations were monitored by the optical density (OD) of ASP-ELP$_{60}$ and ASP-ELP$_{90}$ at 350 nm as a function of temperature. The date was normalized as follows: Normalized OD_{350} = (OD_{sample} - OD_{lowest})/($OD_{highest}$ – OD_{lowest}).

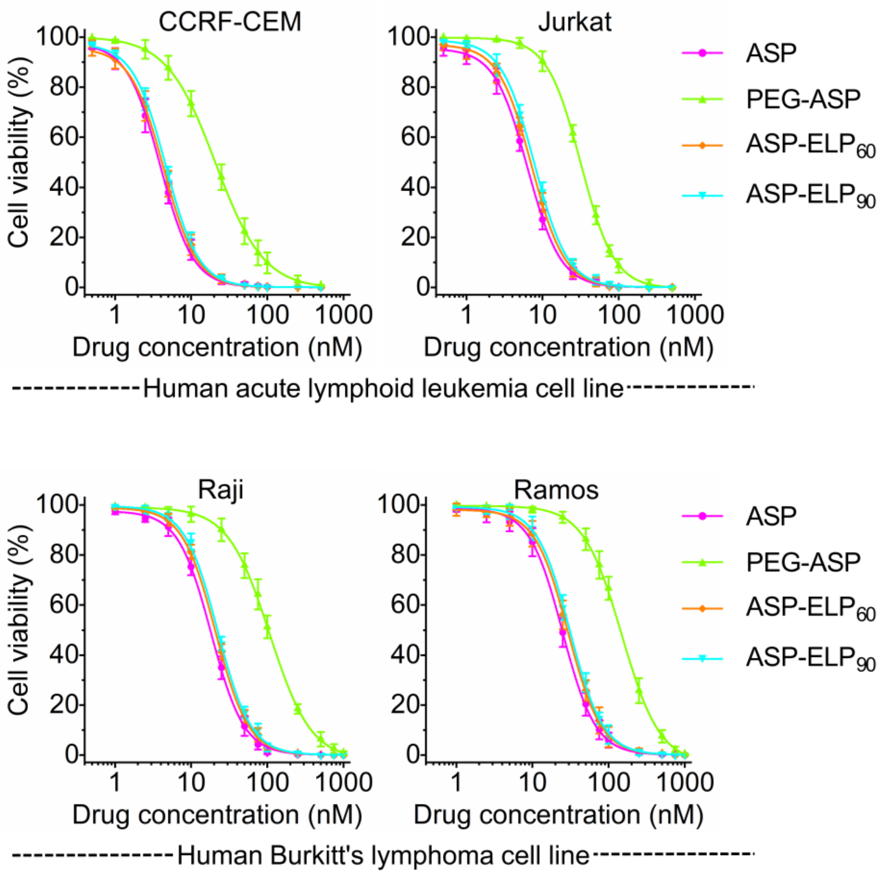

Figure S3. In vitro cytotoxicity of ASP, PEG-ASP, ASP-ELP$_{60}$, and ASP-ELP$_{90}$ on (A) human acute lymphoid leukemia cell lines (Jurkat and CCRF-CEM) and (B) human Burkitt's lymphoma cell lines (Raji and Ramos). The cell viability (%) was calculated as below: (OD_{sample} - $OD_{background}$)/($OD_{control}$ – $OD_{background}$) ×100%. The dose-effect relationship between cell viability and enzymatic concentrations can be fit to sigmoidal curves. Data are mean ± SD (n=3).

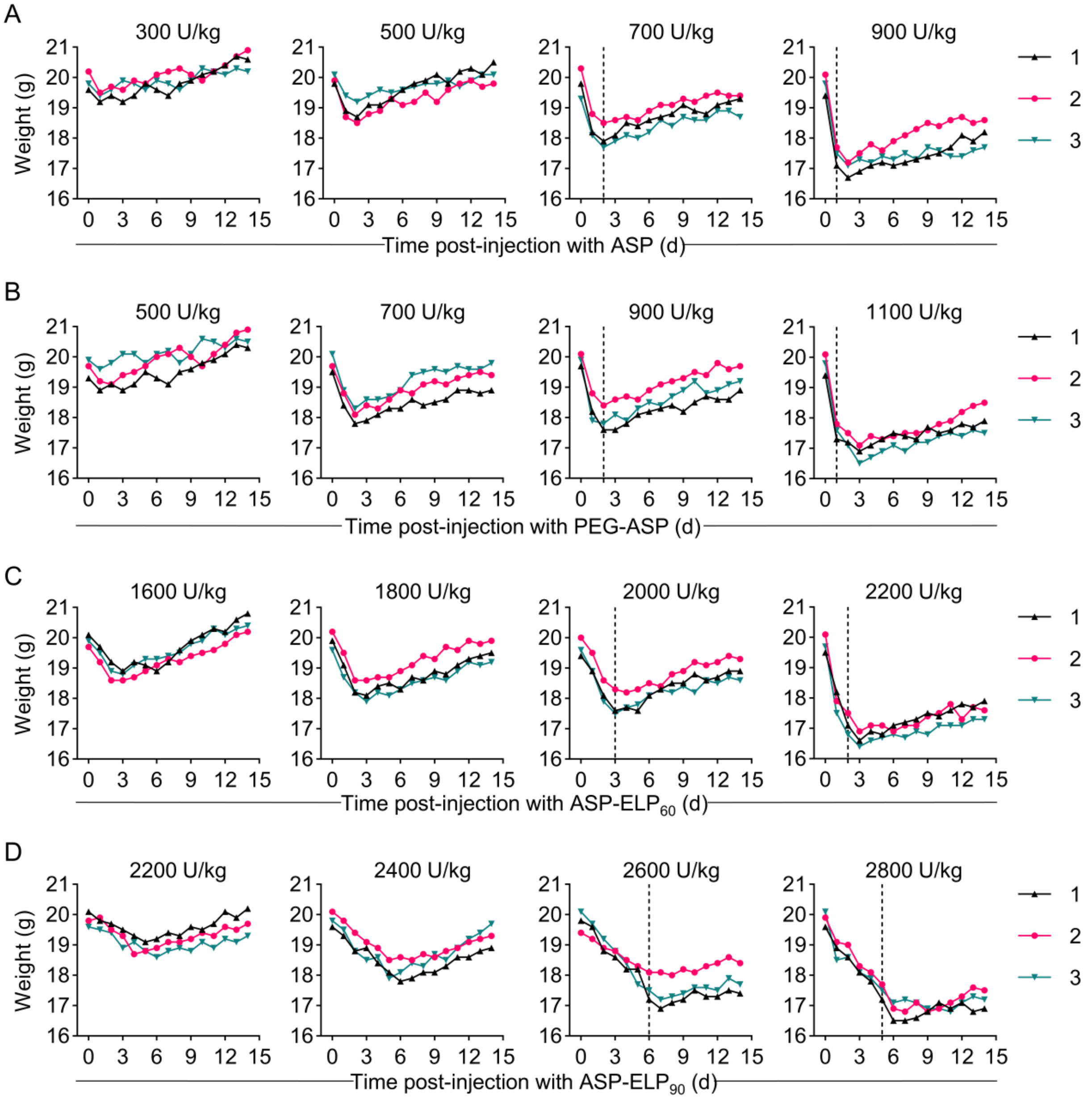

Figure S4. The MTDs of ASP (A), PEG-ASP (B), ASP-ELP$_{60}$ (C), and ASP-ELP$_{90}$ (D) were measured by the dose-escalation trial on healthy BALB/c mice. Dashed vertical lines indicate when the enzymatic dose caused death or weight loss of more than 10% of initial weight in any of the three animals. Data are mean ± SD (n = 3 for each injection).

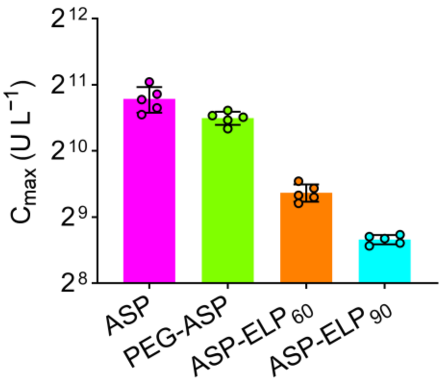

Figure S5. The peak activity concentration (C_{max}) of ASP, PEG-ASP, ASP-ELP$_{60}$, and ASP-ELP$_{90}$. ASP-ELP$_{90}$'s C_{max} was significantly lower than ASP's, PEG-ASP's, and ASP-ELP$_{60}$'s. Data are mean ± SD (n = 5), and the statistical difference was determined by one-way ANOVA followed by Tukey's multiple comparisons test.

References

[1] a)D. E. Meyer, A. Chilkoti, *Nat Biotechnol* **1999**, 17, 1112; b)M. R. Banki, L. Feng, D. W. Wood, *Nat Methods* **2005**, 2, 659; c)W. Y. Wu, C. Mee, F. Califano, R. Banki, D. W. Wood, *Nat Protoc* **2006**, 1, 2257.
